# Characterization of Adult and Pediatric Healthcare-Associated and Community-Associated *Clostridioides difficile* Infections, Canada, 2015–2022 

**DOI:** 10.3201/eid3106.250182

**Published:** 2025-06

**Authors:** Tim Du, Anada Silva, Kelly B. Choi, Cassandra Lybeck, George R. Golding, Romeo Hizon, Sean Ahmed, Nicole Anderson, Suzanne Bakai-Anderson, Blanda Chow, Ian Davis, Meghan Engbretson, Gerald A. Evans, Charles Frenette, Matthew Garrod, Jennie Johnstone, Kevin C. Katz, Pamela Kibsey, Joanne M. Langley, Jenine Leal, Jenna Leamon, Bonita E. Lee, Diane Lee, Yves Longtin, Dominik Mertz, Jessica Minion, Ericka Oates, Michelle Science, Jocelyn A. Srigley, Kathryn N. Suh, Nisha Thampi, Reena Titoria, Kristen Versluys, Alice Wong, Jeannette L. Comeau, Susy S. Hota

**Affiliations:** National Microbiology Laboratory, Winnipeg, Manitoba, Canada (T. Du, G.R. Golding, R. Hizon, S. Ahmed); Public Health Agency of Canada, Ottawa, Ontario, Canada (A. Silva, K.B. Choi, C. Lybeck, D. Lee); Alberta Health Services, Calgary, Alberta, Canada (N. Anderson, B. Chow, J. Leal, E. Oates); Hamilton Health Sciences, Hamilton, Ontario, Canada (S. Bakai-Anderson, D. Mertz); Queen Elizabeth II Health Sciences Centre, Halifax, Nova Scotia, Canada (I. Davis); Children’s Hospital of Eastern Ontario, Ottawa (M. Engbretson, N. Thampi); Kingston Health Sciences Centre, Kingston, Ontario, Canada (G.A. Evans); McGill University Health Centre, Montreal, Quebec, Canada (C. Frenette); Fraser Health Authority, Vancouver, British Columbia, Canada (M. Garrod); Sinai Health, Toronto, Ontario, Canada (J. Johnstone); North York General Hospital, Toronto (K.C. Katz); Royal Jubilee Hospital, Victoria, British Columbia, Canada (P. Kibsey); IWK Health Centre, Halifax (J.M. Langley); Western Health Authority, Corner Brook, Newfoundland, Canada (J. Leamon); Stollery Children’s Hospital, Edmonton, Alberta, Canada (B.E. Lee); Jewish General Hospital, Montreal (Y. Longtin); Regina General Hospital, Regina, Saskatchewan, Canada (J. Minion); The Hospital for Sick Children, Toronto (M. Science); BC Children’s & Women’s Hospitals, Vancouver (J.A. Srigley); The Ottawa Hospital, Ottawa (K.N. Suh); Provincial Health Services Authority, Vancouver (R. Titoria); Interior Health Authority, Kelowna, British Columbia, Canada (K. Versluys); Royal University Hospital, Saskatoon, Saskatchewan, Canada (A. Wong); Dalhousie University, Halifax (J.L. Comeau); University Health Network, Toronto (S.S. Hota)

**Keywords:** Clostridioides difficile, bacteria, enteric infections, adult CDI, antimicrobial resistance, Clostridioides difficile infection, community-associated CDI, healthcare-associated CDI, healthcare-associated infection, nosocomial, pediatric CDI, ribotype, Canada

## Abstract

We investigated epidemiologic and molecular characteristics of healthcare-associated (HA) and community-associated (CA) *Clostridioides difficile* infection (CDI) among adult and pediatric patients in Canadian Nosocomial Infection Surveillance Program hospitals during 2015–2022. Of 30,824 reported CDI cases, 94.9% (29,250/30,824) were among adult (73.2% HA; 26.8% CA) and 5.1% (1,574/30,824) pediatric (77.6% HA; 22.4% CA) patients. During the study period, adult HA CDI rates decreased by 19.9% and CA CDI rates remained stable; pediatric HA CDI rates decreased by 29.6% and CA CDI decreased by 58.3%. Ribotype (RT) 106 was most common among both groups and replaced RT027 as the predominant strain type. RT027 was most associated with adult patients, HA acquisition, severe CDI, and severe outcomes. Moxifloxacin resistance was higher in adult than pediatric cases; clindamycin and rifampin resistance rates were similar between groups. Continued national surveillance is integral to understanding the epidemiology of adult and pediatric CDI in Canada and informing prevention efforts.

*Clostridioides difficile*, a gram-positive, spore-forming anaerobe, is the leading cause of healthcare-associated (HA) diarrhea in high-income countries (*1*). Disease manifestations can range from asymptomatic colonization to pseudomembranous colitis, toxic megacolon, and death (*2*). In the past 2 decades, *C. difficile* has become a major public health concern; the Public Health Agency of Canada and the US Centers for Disease Control and Prevention declared it an urgent health threat in 2019 (*3*,*4*). Healthcare costs for treating *C. difficile* infection (CDI) are substantial, and recurrent episodes further complicate case management (*5*,*6*).

Although some countries have reported increased incidence of HA or community-associated (CA) CDI, the paucity of global data primarily focuses on CDI in adult rather than pediatric populations (*7*,*8*). Studies suggest that pediatric CDI is more likely to be community-associated and have rapid onset and shorter and less complicated infections, whereas illness in adults is characterized by more complicated and severe disease, increased recurrence rates because of more underlying conditions, and risk for infection with hypervirulent strain NAP1/027/BI (*9*,*10*).

Here, we contrast findings of adult and pediatric HA and CA CDI identified in a multicenter study in Canada evaluating incidence, patient characteristics, outcomes, ribotype (RT) prevalence, and antimicrobial resistance during 2015–2022. We also evaluate associations between predominant *C. difficile* RTs and all-cause and CDI-attributable deaths.

## Methods

### Data Sources and Collection

Hospitals participating in the Canadian Nosocomial Infection Surveillance Program (CNISP) have conducted prospective surveillance for HA CDI in hospitalized patients in Canada since 2007 and CA CDI since 2015. By 2022, CNISP encompassed a network of 88 acute-care hospitals across 10 provinces and 1 territory, representing 35% of all acute-care beds in Canada (*11*). We analyzed data collected during 2015–2022 from adult, pediatric, and mixed (adult and pediatric) hospitals. Stool samples, severity indicators, and outcomes were collected during a 2-month targeted surveillance period (March–April) each year for adult patients and year-round for pediatric patients. We included adult and pediatric patients from mixed hospitals in age-specific CDI rate calculations if age-specific denominators were available. Data were collected through the Canadian Network for Public Health Intelligence platform; we verified clinical and laboratory surveillance data to ensure accuracy, as previously described (*12*).

### Case Definition

We used previously described case definitions for primary CDI (*13*). We defined HA CDI as laboratory confirmation of CDI accompanied by compatible clinical symptoms developing >72 hours after admission, or <72 hours after admission if the patient had a previous hospital admission and was discharged within the previous 4 weeks. We defined CA CDI as onset of CDI symptoms <72 hours after admission with no history of hospitalization or healthcare exposure, including outpatient healthcare exposures, within the previous 12 weeks.

A severe CDI case was an albumin level <30 g/L, leukocyte count >15 × 10^9^/L, or both. Severe outcomes were CDI-attributable admission to an intensive care unit (ICU), colectomy, or death <30 days after first *C. difficile*–positive specimen, where CDI was the cause of or contributed to death. All deaths were reviewed by an infectious disease physician or medical microbiologist to determine whether deaths were CDI-attributable, as defined in our published protocol (*14*).

### Laboratory Methods

Hospitals sent stool samples to the National Microbiology Laboratory (NML; Winnipeg, MB, Canada) for *C. difficile* isolation and molecular characterization. We performed *C. difficile* isolation by using an ethanol shock treatment, then selection on *C. difficile* Moxalactam Norfloxacin agar (Oxoid, https://www.oxoid.com) (*15*,*16*). We prepared DNA for PCR analysis and ribotyping by using InstaGene Matrix (Bio-Rad Laboratories, https://www.bio-rad.com) (*16*). We performed multiplex PCR targeting toxin A (*tcdA*), toxin B (*tcdB*), binary toxin (*cdtB*), negative regulator of toxin production (*tcdC*), and triose phosphate isomerase (*tpi*) housekeeping genes (*17*). We performed capillary gel electrophoresis–based ribotyping and RT assignment as previously described (*18*,*19*).

We used Etest strips (bioMérieux, https://www.biomerieux.com) to perform susceptibility testing for metronidazole, clindamycin, vancomycin, rifampin, moxifloxacin, and tigecycline, as previously described (*16*,*20*). We interpreted antimicrobial resistance in accordance with published guidelines (*20*).

### Statistical Analysis

We calculated HA CDI incidence rates as number of cases per 10,000 patient-days and CA CDI incidence rates as number of cases per 1,000 patient admissions. We conducted a sensitivity analysis, restricting our analysis to hospitals participating in the entire 8-year surveillance period. We used the Cochran-Armitage test for categorical variables and the Mann-Kendall test for continuous variables to assess statistically significant trends over time for patient characteristics and outcome results. To compare characteristics between adult and pediatric patients, we used χ^2^ test for categorical variables and Student *t-*test or Wilcoxon rank-sum test for continuous variables. Denominators for individual case characteristics vary because we excluded missing or unknown values from the analysis.

We used multivariable logistic regression to model factors associated with select CDI strains (RT027 and RT106) and a severe CDI outcome and adjusted for a priori–selected confounders of age group, sex, severe CDI, and CDI case type (i.e., HA vs. CA). We used 2-tailed statistical tests and considered p<0.05 significant. We performed all analyses in R version 4.3.0 (The R Project for Statistical Computing, https://www.r-project.org).

## Results

The study encompassed 30,824 inpatient cases of primary CDI from CNISP during 2015–2022. Adult CDI accounted for 94.9% (n = 29,250) and pediatric CDI for 5.1% (n = 1,574) of cases. Among adult patients, HA CDI accounted for 73% (n = 21,405) and CA CDI for 27% (n = 7,845) of cases. Among pediatric patients, HA CDI accounted for 78% (n = 1,222) and CA CDI for 22% (n = 352) of cases. Hospital participation varied by age group and case type throughout the study period (Appendix 1 Table 1).

**Figure 1:**
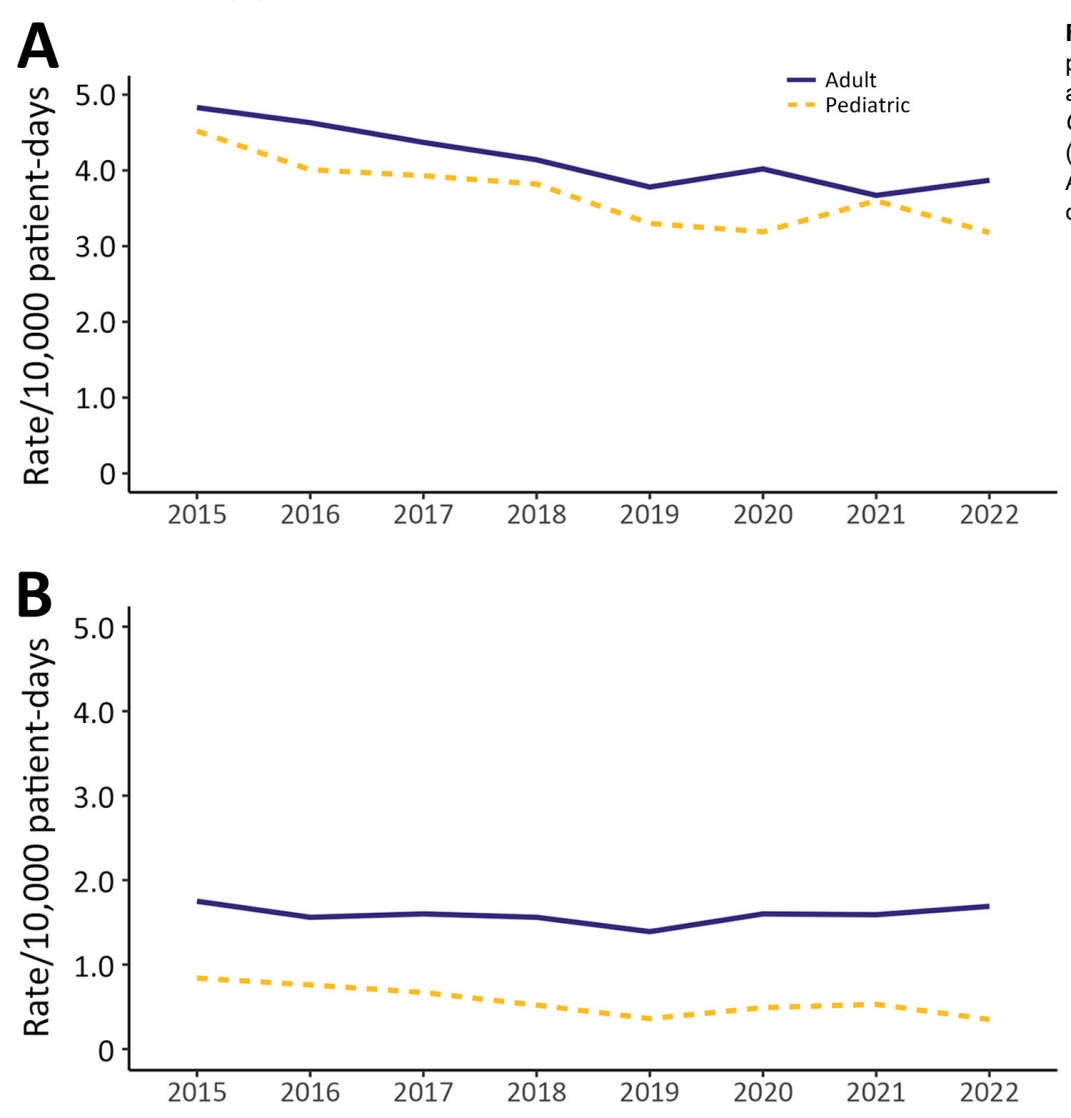
National adult and pediatric healthcare-associated and community-associated *Clostridioides difficile* infection (CDI) rates, Canada, 2015–2022. A) Healthcare-associated CDI; B) community-associated CDI.

During 2015–2022, adult HA CDI rates decreased by 19.9%, from 4.83 to 3.87 cases/10,000 patient-days (p = 0.006), whereas CA CDI rates remained stable, ranging from 1.39 to 1.75 cases/1,000 admissions. Pediatric HA CDI rates decreased by 29.6%, from 4.52 to 3.18 cases/10,000 patient-days (p = 0.003), and CA CDI rates decreased by 58.3%, from 0.84 to 0.35 cases/1,000 admissions (p = 0.0133) (Figure 1).

**Figure 2:**
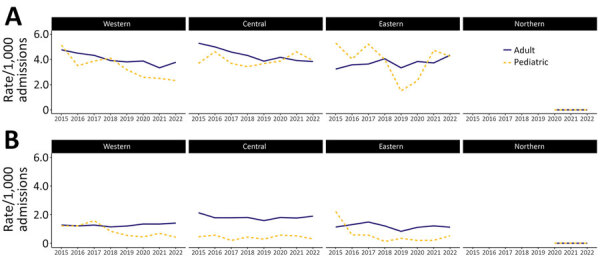
Regional adult and pediatric healthcare-associated and community-associated *Clostridioides difficile* infection (CDI) rates, Canada, 2015–2022. A) Healthcare-associated CDI; B) community-associated CDI. Western region includes British Columbia, Alberta, Saskatchewan, and Manitoba; Central region includes Ontario and Quebec; Eastern region includes Nova Scotia, New Brunswick, Prince Edward Island, and Newfoundland and Labrador. Northern region is Nunavut. The Northern region has reported 0 cases of CDI since they started conducting surveillance in 2020.

Regionally, adult HA CDI rates decreased significantly in the central (27.4%; p = 0.003) and western (20.8%; p = 0.003) regions of Canada, but rates increased by 34% in the eastern region (p = 0.0478) (Figure 2). Pediatric HA CDI rates significantly decreased by 54.7% (p = 0.0065) in the western region, but central and eastern region rates fluctuated. Pediatric CA CDI rates decreased significantly by 65.3% (p = 0.0178) in the western region, but eastern region rates fluctuated from 0.19 to 0.58 cases/1,000 admissions after an initial decrease from 2.22 cases/1,000 admissions in 2015. Sensitivity analyses that restricted analysis to hospitals participating for the entire study period by region for adult and pediatric surveillance and those that conducted both HA and CA CDI surveillance yielded no statistically significant differences (data not shown).

### Clinical Manifestations

We aggregated patient characteristics and outcomes by age group (adult and pediatric) and case type (Tables 1, 2). For HA CDI, fewer pediatric than adult patients were female (45% vs. 49%; p = 0.024), but differences between pediatric and adult CA CDI were not statistically significant for female sex (49% vs. 55%; p = 0.07). Adults with HA CDI were significantly older than adults with CA CDI (70 [interquartile range (IQR) 59–81] vs. 67 [IQR 54–78] years; p*<*0.0001). Pediatric patients with CA CDI were older than those with HA CDI (10 [IQR 3–13] vs. 7 [IQR 4–15] years; p*=*0.0007). For HA CDI, the median days from admission to infection were longer in adult (10 [IQR 5–21] days) than pediatric patients (7 [IQR 2–15] days) (p<0.001). Adult patients experienced significantly longer hospital stays for HA CDI than did pediatric patients (15 [IQR 9–24] vs. 9 [IQR 5–18] days for HA CDI, 7 [IQR 4–13] vs. 5 [IQR 2–8] days for CA CDI; p<0.001 for both). Regardless of acquisition type, metronidazole was the drug most used to treat CDI among pediatric patients and vancomycin was most common among adult patients. Fecal microbiota transplantation referral was uncommon among adult and pediatric patients.

**Table 1:** Characteristics of adult and pediatric healthcare-associated *Clostridioides difficile* infections, Canada, 2015–2022*

Characteristics	Overall, n = 22,627	Adult cases, n = 21,405	Pediatric cases, n = 1,222	p value†
Median age, y (IQR)	69 (56–80)	70 (59–81)	7 (3–13)	
Sex				**0.024**
F	10,994/22,625 (49)	10,445/21,404 (49)	549/1,221 (45)	
M	11,631/22,625 (51)	10,959/21,404 (51)	672/1,221 (55)	
Median days from admission to infection (IQR)	10 (4–21)	10 (5–21)	7 (2–15)	**<0.001**
Median length of stay, d (IQR)	14 (7–23)	15 (9–24)	9 (5–18)	**<0.001**
Treatment				NA
Metronidazole	6,377/18,907 (34)	5,818/17,947 (32)	559/960 (58)	
Vancomycin	10,876/18,907 (58)	10,588/17,947 (59)	288/960 (30)	
Metronidazole and vancomycin	982/18,907 (5.2)	957/17,947 (5.3)	25/960 (2.6)	
Fidaxomicin	22/18,907 (0.1)	22/17,947 (0.1)	0/960 (0)	
Other	186/18,907 (1.0)	170/17,947 (0.9)	16/960 (1.7)	
No treatment	464/18,907 (2.5)	392/17,947 (2.2)	72/960 (7.5)	
FMT referral	17/9,825 (0.2)	17/9,318 (0.2)	0/507 (0)	>0.9
30-day outcomes‡				
Loop ileostomy§	14/2,413 (0.6)	14/1,816 (0.8)	0/597 (0)	**0.028**
All-cause mortality	416/4,429 (9.4)	399/3,454 (12)	17/975 (1.7)	**<0.001**
Severe outcome¶	205/4,232 (4.8)	180/3,270 (5.5)	25/962 (2.6)	**<0.001**
ICU admission for CDI complications	73/4,448 (1.6)	61/3,477 (1.8)	12/971 (1.2)	0.3
Colectomy	62/4,296 (1.4)	50/3,329 (1.5)	12/967 (1.2)	0.5
CDI-attributable death	99/405 (24)	98/388 (25)	1/17 (5.9)	0.084
CDI recurrence#				
Recurrence	223/2,709 (8.2)	205/2,514 (8.2)	18/195 (9.2)	0.6
Median days from primary infection to recurrence (IQR)	29 (21–40)	29 (21–41)	26 (22–30)	0.4
Recurrence length of stay, d (IQR)	10 (6–19)	10 (6–16)	30 (30–30)	0.2
Recurrence FMT referral	0/81	0/79	0/2	NA
Recurrence loop ileostomy	1/81 (1.2)	1/78 (1.3)	0/3	>0.9
Recurrence all-cause mortality	16/189 (8.5)	16/177 (9.0)	0/12	0.6
Recurrence severe outcome¶	8/186 (4.3)	8/174 (4.6)	0/12	>0.9
ICU admission for recurrent CDI complications	3/198 (1.5)	3/185 (1.6)	0/13	>0.9
Recurrence-attributable death	5/16 (31)	5/16 (31)	0	NA
Recurrence colectomy	1/196 (0.5)	1/183 (0.5)	0/13	>0.9

*Values are no. cases/no. in category (%) except as indicated. Denominators for individual case characteristic vary because missing or unknown values were excluded from the analysis. Bold font indicates statistical significance. CDI, *C. difficile* infection; FMT, fecal microbiota transplant; ICU, intensive care unit; IQR, interquartile range; NA, not applicable. †p value determined by Wilcoxon rank-sum test, Fisher exact test, or Pearson χ^2^ test. ‡Outcome data were collected during March–April of each calendar year for adult patients and year-round for pediatric patients. §Loop ileostomy added in 2018. ¶Severe outcome was defined as CDI-attributable admission to an intensive care unit, colectomy, or death within 30 d of positive *C. difficile* specimen where CDI was the cause of death or contributed to death. #Adult and pediatric cases identified during March–April of each calendar year were followed prospectively for 8 weeks for recurrence.

**Table 2:** Characteristics of adult and pediatric community-associated *Clostridioides difficile* infections, Canada, 2015–2022*

Characteristics	Overall, n = 8,197	Adult cases, n = 7,845	Pediatric cases, n = 352	p value†
Median age, y (IQR)	66 (52–78)	67 (54–79)	10 (4–15)	
Sex				
F	4,525/8,197 (55)	4,351/7,845 (55)	174/352 (49)	0.070
M	3,672/8,197 (45)	3,494/7,845 (45)	178/352 (51)	
Median length of stay, d (IQR)	7 (4, 12)	7 (4, 13)	5 (2, 8)	**<0.001**
Treatment				NA
Metronidazole	2,264/7,497 (30)	2,100/7,225 (29)	164/272 (60)	
Vancomycin	4,515/7,497 (60)	4,441/7,225 (61)	74/272 (27)	
Metronidazole and vancomycin	448/7,497 (6.0)	436/7,225 (6.0)	12/272 (4.4)	
Fidaxomicin	7/7,497 (<0.1)	7/7,225 (<0.1)	0/272	
Other	72/7,497 (1.0)	72/7,225 (1.0)	0/272	
No treatment	191/7,497 (2.5)	169/7,225 (2.3)	22/272 (8.1)	
FMT referral	13/4,360 (0.3)	13/4,228 (0.3)	0/132	>0.9
30-day outcomes‡				
Loop ileostomy§	8/874 (0.9)	8/730 (1.1)	0/144	0.4
All-cause mortality	5/1,473 (6.4)	95/1,201 (7.9)	0/272	**<0.001**
Severe outcome¶	71/1,454 (4.9)	62/1,184 (5.2)	9/270 (3.3)	0.2
ICU admission for CDI complications	27/1,482 (1.8)	22/1,203 (1.8)	5/279 (1.8)	>0.9
Colectomy	23/1,470 (1.6)	19/1,193 (1.6)	4/277 (1.4)	>0.9
CDI-attributable death	30/89 (34)	30/89 (34)	0	NA
CDI recurrence#				
Recurrence	81/1,149 (7.0)	79/1,065 (7.4)	2/84 (2.4)	0.083
Median days from primary infection to recurrence (IQR)	29 (23–38)	29 (23–38)	36 (36–36)	0.5
Recurrence length of stay, d (IQR)	9 (5, 17)	9 (5, 17)	NA	NA
Recurrence FMT referral	0/35	0/34	0/1	NA
Recurrence loop ileostomy	0/37	0/36	0/1	NA
Recurrence all-cause mortality	3/67 (4.5)	3/66 (4.5)	0/1	>0.9
Recurrence severe outcome¶	1/61 (1.6)	1/61 (1.6)	0	NA
ICU admission for recurrent CDI complications	0/69	0/69	0	NA
Recurrence-attributable death	1/2 (50)	1/2 (50)	0	NA
Recurrence colectomy	0/68	0/67	0/1	NA

*Values are no. cases/no. in category (%) except as indicated. Denominators for individual case characteristic vary because missing or unknown values were excluded from the analysis. Bold font indicates statistical significance. CDI, *C. difficile* infection; FMT, fecal microbiota transplant; ICU, intensive care unit; IQR, interquartile range; NA, not applicable. †p value determined by Wilcoxon rank-sum test, Fisher exact test, or Pearson χ^2^ test. ‡Outcome data was collected during March–April of each calendar year for adult patients and year-round for pediatric patients. §Loop ileostomy added in 2018. ¶Severe outcome was defined as CDI-attributable admission to an intensive care unit, colectomy, or death within 30 d of positive *C. difficile* specimen where CDI was the cause of death or contributed to death. #Adult and pediatric cases identified during March–April of each calendar year were followed prospectively for 8 weeks for recurrence.

### Ribotyping Analysis

Of 30,824 cases with linked epidemiologic data, NML successfully characterized 4,622 (3,560 adult, 1,062 pediatric) samples that met study criteria. Of 3,560 adult samples analyzed, 74.7% (2,659/3,560) were HA CDI and 25.3% (901/3,560) were CA CDI, and we identified 241 unique RTs.

**Figure 3:**
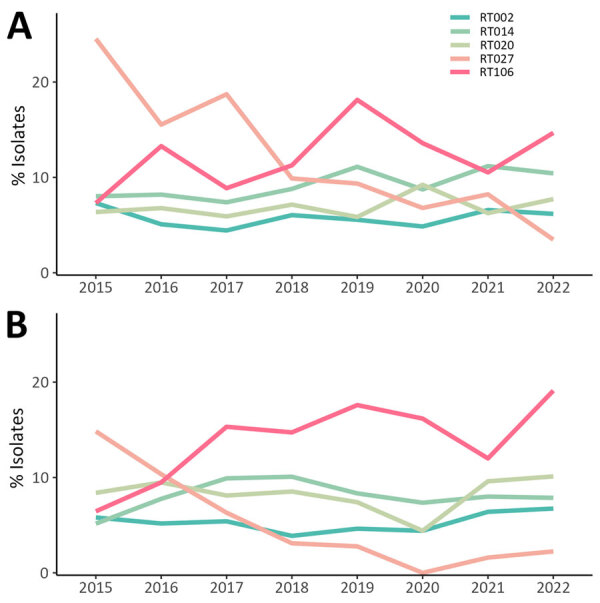
Percentages of 5 most common ribotypes detected among isolates in a characterization of adult healthcare-associated and community-associated *Clostridioides difficile* infection (CDI), Canada, 2015–2022. A) Healthcare-associated CDI rates; B) community-associated CDI rates. RT, ribotype.

The most common adult RTs were RT106 (11.9% HA, 13.2% CA), RT027 (13.2% HA, 5.9% CA), RT014 (9.1% HA, 8.0% CA), RT020 (6.7% HA, 8.4% CA), and RT002 (5.8% HA, 5.3% CA) (Figure 3). The 20 most common adult CDI RTs accounted for 73.9% of isolates tested (Appendix 1 Figure 1). Among adult CDI cases, RT027 rates decreased from 21.9% in 2015 to 3.2% in 2022 (p = 0.003). RT106 rates fluctuated over the study period but increased overall from 7.1% in 2015 to 15.8% in 2022. During 2015–2022, RT106 replaced RT027 as the predominant strain type and had an overall combined prevalence of 12.2% (434/3,560).

**Figure 4:**
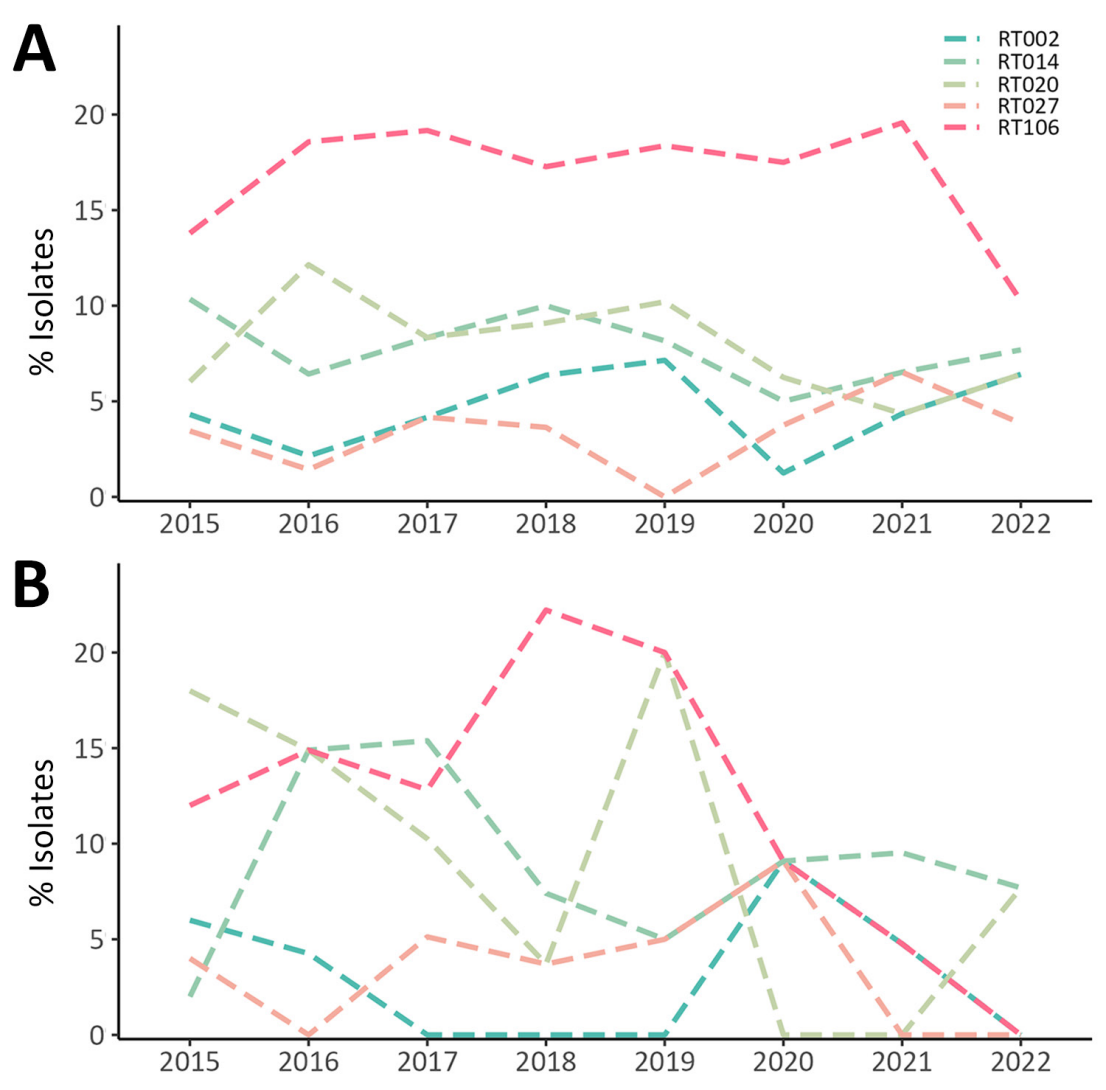
Percentages of 5 most common RTs detected among isolates in a characterization of pediatric healthcare-associated and community-associated *Clostridioides difficile* infection (CDI), Canada, 2015–2022. A) Healthcare-associated CDI rates; B) community-associated CDI rates. RT, ribotype.

Of 1,062 pediatric samples analyzed, 834 (78.5%) were HA CDI and 228 (21.5%) were CA CDI, and we identified 145 unique RTs. The most common RTs among pediatric CDI cases were RT106 (17.0% HA, 13.2% CA), RT020 (8.2% HA, 11.4% CA), RT014 (7.9% HA, 9.2% CA), RT056 (4.4% HA, 4.0% CA), and RT002 (4.4% HA, 3.1% CA) (Figure 4). The 20 most prevalent pediatric RTs accounted for 74.6% of isolates tested (Appendix 1 Figure 2). RT106 rates increased from 13.3% in 2015 to 16.8% in 2021 and decreased to 8.8% in 2022 (p = 0.02). In contrast to adult cases, RT027 prevalence was lower in pediatric cases (average 3.2%) and fluctuated throughout the study period.

Prevalence of livestock-associated strains RT078 and RT126 averaged 2.8% (range 1.8%–5.8%) among adult CDI cases. Among pediatric CDI cases, those strains averaged 2.1% (range 1.1%–3.8%) (Appendix 1 Table 2).

### Clinical Outcomes

Of the 30,824 cases analyzed, 19.1% (n = 5,902) had outcome data available for adult (n = 4,715) and pediatric (n = 1,265) patients. Overall, the 30-day all-cause mortality rate was 10.6% (494/4,655) for adult patients, and 26.8% (128/477) of those deaths were directly or indirectly attributable to CDI. HA CDI comprised most adult all-cause (80.8%; 399/494) and CDI-attributable (76.6%; 98/128) deaths. We noted no major differences in adult CDI-attributable death by sex or older age (data not shown). In contrast, 1.4% (17/1,247) of pediatric CDI patients died of any cause at 30 days, and only 1 death was CDI-attributable and was HA CDI. We noted no statistically significant differences in all-cause death by age group (1 to <2 years of age: 2.9% [4/139]; 2 to <12 years of age: 1.2% [8/676]; 12 to <18 years of age: 1.2% [5/432]; p = 0.3) (data not shown).

Comparing acquisition types, pediatric patients had significantly lower all-cause mortality rates than adult patients for HA CDI (1.7% vs. 12%; p<0.001) and CA CDI (0 vs. 7.9%; p<0.001) (Tables 1, 2). We observed no significant differences in ICU admission resulting from CDI complications between adult and pediatric patients for either acquisition type.

Of 5,686 cases with available data, 5.4% (242/4,454) of adult cases and 2.8% (34/1,232) of pediatric cases had a severe CDI-related outcome <30 days after the first *C. difficile*–positive specimen. More adult than pediatric patients experienced a severe outcome for HA CDI (12% vs. 1.7%; p<0.001), but we noted no statistically significant difference for CA CDI (5.2% adult vs. 3.3% pediatric; p = 0.2) (Tables 1, 2).

Compared with patients with non-RT027 CDI, patients with RT027 had adjusted odds of 2.15 (95% CI 1.63–2.90; p<0.0001) times higher for severe CDI and of 1.93 (95% CI 1.31–2.90; p = 0.0005) times higher for CDI-related severe outcome (Table 3). In addition, RT027 patients had much higher odds of being adult than pediatric and of having HA rather than CA CDI. In contrast, RT106 strains were less likely to be associated with adult cases, but we found no evidence of association with severe CDI outcomes compared with non-RT106 strains.

**Table 3:** Multivariate regression modeling of epidemiologic factors in characterization of adult and pediatric healthcare-associated and community-associated *Clostridioides difficile* infections, Canada, 2015–2022*

Characteristics	Univariable analysis			Multivariable analysis	
	Odds ratio (95% CI)	p value		Odds ratio (95% CI)	p value
RT027 (n = 438) vs. non-RT027 (n = 4,184) strains					
Age group					
Adult	**3.87 (2.75–5.63)**	**<0.0001**		**3.42 (2.35–5.18)**	**<0.0001**
Pediatric	Referent			Referent	
Sex					
F	Referent			Referent	
M	0.97 (0.79–1.17)	0.720		0.97 (0.79–1.20)	0.970
CDI case type					
Community-associated	Referent			Referent	
Healthcare-associated	**2.16 (1.65–2.89)**	**<0.0001**		**2.18 (1.64–2.95)**	**<0.0001**
Severe CDI†	**2.24 (1.71–2.99)**	**<0.0001**		**2.15 (1.63–2.90)**	**<0.0001**
Severe outcome‡	**2.27 (1.56–3.24)**	**<0.0001**		**1.93 (1.31–2.90)**	**0.0005**
RT106 (n = 606) vs. non-RT106 (n = 4,016) strains					
Age group					
Adult	**0.72 (0.59–0.87)**	**0.0007**		**0.71 (0.58–0.88)**	**0.002**
Pediatric	Referent			Referent	
Sex					
F	Referent			Referent	
M	0.905 (0.76–1.07)	0.251		0.89 (0.74–1.07)	0.208
CDI case type					
Community-associated	Referent			Referent	
Healthcare-associated	0.99 (0.81–1.21)	0.921		1.01 (0.82–1.25)	0.936
Severe CDI†	0.83 (0.69–1.01)	0.0621		0.87 (0.72 – 1.06)	0.168
Severe outcome‡	0.76 (0.41–1.17)	0.233		0.80 (0.50 – 1.24)	0.345

*Bold font indicates statistical significance. CDI, *C. difficile* infection. †Severe CDI defined as albumin level <30 g/L, leukocyte count >15 × 10^9^ cells/L, or both. ‡Severe outcome was defined as CDI-attributable admission to an intensive care unit, colectomy, or death within 30 d of first positive *C. difficile* specimen where CDI was the cause of death or contributed to death.

### CDI Recurrence

Rates of recurrent CDI within 8 weeks of the first positive specimen were similar in adult (7.9%) and pediatric (7.2%) patients (p = 0.6). Median time from primary infection to recurrence was 29 (IQR 22–40) days, and we noted no major difference between adult and pediatric patients (data not shown). We noted no statistically significant differences in recurrence by age group **(**Tables 1, 2); however, pediatric recurrence was significantly higher for HA CDI (9.2%; 18/195) than CA CDI (2.4%; 2/84) (p = 0.04), and adult recurrence was similar, 8.2% (205/2,514) for HA and 7.4% (79/1,065) for CA (p = 0.5) (data not shown). Among cases with recurrent outcome data (n = 247), 9 (3.6%) had severe outcomes; all were adult patients.

### Antimicrobial Susceptibility

We conducted antimicrobial resistance testing for isolates collected during 2015–2022 (Appendix 2 Tables 1, 2). Among HA CDI during the study years, 18.0% of adult and 5.8% of pediatric cases were moxifloxacin resistant, 27.7% of adult and 24.0% of pediatric cases were clindamycin resistant, and 1.6% of adult and 1.7% of pediatric cases were rifampin resistant. For CA CDI, 10.7% of adult and 7.9% of pediatric cases were moxifloxacin resistant, 28.4% of adult and 23.7% of pediatric cases were clindamycin resistant, and 1.3% of adult and no pediatric cases were rifampin resistant. Overall moxifloxacin resistance was higher in adult (16.2%) than pediatric (6.2%) populations. Of note, from 2015 to 2022, moxifloxacin resistance decreased by 27.3% for adult HA CDI and 14.2% for adult CA CDI. Despite variability in clindamycin resistance (range 8.8%–50.4%) during the study period, overall resistance was 27.9% for adult and 23.9% for pediatric cases.

Among the isolates examined, RT027 accounted for 55.0% (316/575) of adult and 24.2% (16/66) of pediatric moxifloxacin-resistant isolates. Among RT027 samples, 78.2% (316/404) of adult and 47.1% (16/34) of pediatric isolates were moxifloxacin resistant. Among moxifloxacin-resistant RT027, all pediatric (n = 16) and 97.5% (308/316) of adult samples had MICs >32 µg/mL. In contrast, RT106, the most prevalent (13.1%) pediatric strain type and second most prevalent (12.2%) adult strain type, accounted for 16.7% (11/66) of pediatric and 6.6% (38/575) adult moxifloxacin-resistant isolates. Overall, fluoroquinolone resistance in RT106 isolates was much lower in adult (8.8%, 38/434) and pediatric (6.4%, 11/172) populations.

Of note, multidrug resistance was more common among tested RT027 strains. Of 231 (206 adult, 25 pediatric) isolates resistant to both moxifloxacin and clindamycin, 37.2% (n = 86; 83 adult, 3 pediatric) were RT027. Of 31 (29 adult, 2 pediatric) isolates resistant to moxifloxacin, clindamycin, and rifampin, 51.6% (n = 16) were RT027. In contrast, no RT106 isolates exhibited resistance to moxifloxacin, clindamycin, and rifampin.

One RT012 isolate from a 2018 pediatric HA CDI case was metronidazole resistant (MIC 48 µg/mL), and 2 adult cases were vancomycin intermediate resistant: an RT002 HA CDI case in 2019 (MIC 6 µg/mL) and an RT126 CA CDI case in 2021 (MIC 12 µg/mL). Treatment and outcome data were not available for the metronidazole-resistant case. For the 2 adult vancomycin intermediate resistant cases, both were treated with vancomycin and had no severe outcomes, indicated treatment failure, or reported recurrence. We did not observe tigecycline resistance in any cases during the study period.

## Discussion

We analyzed 8 years of CDI surveillance data from adult and pediatric inpatients from acute care hospitals in Canada. Nationally, HA CDI rates declined by 19.9% in adult and 29.6% in pediatric inpatients and CA CDI rates declined by 58.3% among pediatric inpatients. Epidemiologic and molecular characterization of CDI in adult and pediatric populations revealed more severe 30-day outcomes among adult than pediatric patients and that RT106 was the predominant ribotype in both populations.

Decreasing national adult and pediatric HA CDI rates coincided with global declines in CDI, including in the United States (*21*,*22*). One study reported the annual rate of pediatric CDI-associated hospitalization in the United States increased from 7.24 to 12.8/10,000 hospitalizations during 1997–2006 (*23*), and another reported a doubling in annual incidence among 22 pediatric hospitals in a multicenter study during 2001–2006 (*24*). A more recent estimate showed the overall CDI burden in the United States decreased 24% from 2011 to 2017, after adjusting for testing method (*22*). The decreases in pediatric HA CDI rates we report during 2015–2022 are a reversal from trends reported by CNISP from 2007–2011 (*25*), and adult HA CDI rates continue the decline trends observed during 2011–2016 (*19*). Although the reasons for declining incidence in Canada are not fully elucidated, enhanced infection control and antimicrobial stewardship measures might have contributed (*26*,*27*). Declines in RT027 and changes in testing methodology and criteria might have further contributed to decreased CDI rates (*28*).

The nondecreasing trends in adult CA CDI incidence during 2015–2022 could be attributed to the increase in adult CA CDI during the COVID-19 pandemic period (2019­–2022), after our previous report of declining incidence during 2015–2019 (*17*). In agreement with previous findings, our study showed that adult CA CDI patients were more likely to be younger and female compared with HA CDI patients (*29*–*31*). In contrast, pediatric CA CDI patients were more likely to be older, and we noted no differences in sex. Although age group–specific data on CA CDI incidence is sparse, population-based estimates in the United States increased during 2012–2017 (*32*). Data from Europe reported a 2018–2020 mean CA CDI hospital incidence of 1.35 cases/1,000 patient admissions (*33*), but those data were not stratified by age group. The decreases in pediatric CDI rates we report coincide with other reported decreases among hospitalized pediatric patients from 49 US pediatric tertiary care centers without distinction of acquisition type (*34*). A single-center study in Taiwan reported an overall 2013–2019 pediatric CA CDI incidence rate of 0.564 cases/1,000 patient admissions, although that rate increased substantially from the 2007–2012 period (*35*). 

Molecular analysis of CDI isolates in Canada revealed a dynamic and heterogeneous RT population and that RT106 predominated in both adult and pediatric patients. RT106, first identified in the United Kingdom in 1999 (*36*), is now found worldwide and is among the most prevalent strains in the United States (*37*). Reports of enhanced spore-producing and biofilm-forming capabilities of RT106 suggest adaptive advantages that might enable greater persistence in the environment and hospital settings, possibly leading to increased infection and recurrence rates (*36*–*39*). 

RT027 prevalence in Canada decreased substantially among adults and remained relatively low in pediatrics inpatients. Parallel decreases in RT027 prevalence have been observed in North America, the United Kingdom, and elsewhere (*1*,*32*,*40*–*42*). Despite a decrease in prevalence, multivariable analysis revealed that patients infected with RT027 were more likely to be adults with HA CDI, who also experienced more severe CDI-related ICU admission, colectomy, or death.

Livestock-associated *C. difficile* RT078 and RT126, notable from a One Health perspective, have demonstrated increased virulence, disease severity, and epidemic potential in several countries in Europe (*43*,*44*) but appear to be uncommon in hospitalized CDI patients in Canada. Overall, adult RT078 and RT126 prevalence was 2.8% and pediatric prevalence was 2.1%, a small increase from previously reported data (*13*).

Antimicrobial susceptibility testing suggested that resistance in *C. difficile* is lower in Canada than in the United States or globally (*8*). The percentage of tested isolates resistant to moxiﬂoxacin (2%–87%), clindamycin (15%–97%), and metronidazole (0.1−18.3%) were previously reported (*8*,*45*). Stratified by case type, HA and CA CDI isolates revealed no major differences in resistance for either adult or pediatric populations, except for moxifloxacin. Adult moxifloxacin resistance was 16.5% for HA CDI and 10.0% for CA CDI. Moxifloxacin resistance was lower in the pediatric population; 5.7% of HA and 7.7% CA CDI isolates exhibited resistance. Most strikingly, among RT027 isolates characterized, moxifloxacin resistance decreased from 92.1% to 9.1% in adult and from 66.7% to 0 in pediatric populations during 2015–2022. The exceptionally high percentage of tested RT027 moxifloxacin-resistant isolates recorded at the beginning of our study parallels rates observed in the United States, where 98% resistance was recorded (*46*). Similarly, our findings are consistent with a previously published study from North America that found RT027 strains are more likely to be resistant to multiple drugs, including clindamycin, moxiﬂoxacin, and rifampin (*47*). The lower resistance observed in our diverse RT population is consistent with the suggestion that RT heterogeneity is inversely correlated with antimicrobial resistance, as measured by cumulative resistance scores (*48*,*49*).

The first limitation of our study is that hospitals self-select whether to participate in both HA and CA CDI surveillance; participation varied over time, which might have influenced comparisons between patients and observed temporal trends. Thus, we conducted sensitivity analyses restricted to hospitals that conducted both HA and CA CDI surveillance and to hospitals that participated in all 8 years of the study period, and results of those analyses were not significantly different from the full analyses. Second, although CDI diagnostic testing methods were collected throughout the study period, data completeness was not consistent from year to year, limiting the inferences we could make regarding the effect of CDI diagnostic testing methods on adult and pediatric rates over time. Third, for CA CDI surveillance, we captured data from patients admitted to a CNISP hospital and requiring medical intervention for CDI symptoms or other underlying conditions. The features and outcomes of those patients might not be relevant to patients with CA CDI who do not require hospital care. Fourth, isolates and outcome data were only collected for adults during a 2-month targeted period and might not represent the epidemiologic and molecular characteristics across the full year. Finally, although a qualified physician determined the cause of death in CDI patients, attribution of death is difficult and could be subjective.

In conclusion, rates of adult and pediatric HA CDI in Canada declined during 2015–2022, coinciding with decreased prevalence of RT027 and increased prevalence of RT106. We noted major decreases in antimicrobial resistance to moxifloxacin in both adult HA and CA CDI populations, concordant with an overall decrease in prevalence of RT027. Despite declining rates, CDI continues to be a major health burden in Canada. To ensure continued success in combatting this global health threat, robust national surveillance and infection prevention and control programs are integral to clarifying CDI epidemiology, investigation, and control.

Appendix 1 Additional information on characterization of adult and pediatric healthcare-associated and community-associated *Clostridioides difficile* infections, Canada, 2015–2022.

Appendix 2 Antimicrobial resistance interpretation for characterization of adult and pediatric healthcare-associated and community-associated *Clostridioides difficile* infections, Canada, 2015–2022.

## References

[1] Katz KC, Golding GR, Choi KB, Pelude L, Amaratunga KR, Taljaard M, et al.; Canadian Nosocomial Infection Surveillance Program. The evolving epidemiology of *Clostridium difficile* infection in Canadian hospitals during a postepidemic period (2009-2015). CMAJ. 2018;190:E758–65. 10.1503/cmaj.180013 29941432 PMC6019340

[2] Rupnik M, Wilcox MH, Gerding DN. *Clostridium difficile* infection: new developments in epidemiology and pathogenesis. Nat Rev Microbiol. 2009;7:526–36. 10.1038/nrmicro2164 19528959

[3] Canadian Antimicrobial Resistance Surveillance System (CARSS). Pan-Canadian action plan on antimicrobial resistance. Ottowa (ON): Public Health Agency of Canada; 2023.

[4] Centers for Disease Control and Prevention. Antibiotic resistance threats in the United States, 2019. Atlanta: The Centers; 2019.

[5] Bouza E. Consequences of *Clostridium difficile* infection: understanding the healthcare burden. Clin Microbiol Infect. 2012;18(Suppl 6):5–12. 10.1111/1469-0691.12064 23121549

[6] Levy AR, Szabo SM, Lozano-Ortega G, Lloyd-Smith E, Leung V, Lawrence R, et al. Incidence and costs of *Clostridium difficile* infections in Canada. Open Forum Infect Dis. 2015;2:ofv076. 10.1093/ofid/ofv076 26191534 PMC4503917

[7] Eng L, Collins DA, Alene KA, Bory S, Theng Y, Vann P, et al. *Clostridioides* (*Clostridium*) *difficile* infection in hospitalized adult patients in Cambodia. Microbiol Spectr. 2025;13:e0274724. 10.1128/spectrum.02747-24 39969191 PMC11960136

[8] Zhao H, Nickle DC, Zeng Z, Law PYT, Wilcox MH, Chen L, et al. Global landscape of *Clostridioides difficile* phylogeography, antibiotic susceptibility, and toxin polymorphisms by post-hoc whole-genome sequencing from the MODIFY I/II studies. Infect Dis Ther. 2021;10:853–70. 10.1007/s40121-021-00426-6 33751421 PMC8116447

[9] McFarland LV, Ozen M, Dinleyici EC, Goh S. Comparison of pediatric and adult antibiotic-associated diarrhea and *Clostridium difficile* infections. World J Gastroenterol. 2016;22:3078–104. 10.3748/wjg.v22.i11.3078 27003987 PMC4789985

[10] Silva A, Du T, Choi KB, Pelude L, Golding GR, Hizon R, et al.; CNISP C. difficile working group. CNISP C. difficile working group. Epidemiology of primary and recurrent healthcare-associated and community-associated pediatric *Clostridioides difficile* infection in Canada, 2015–2020. J Pediatric Infect Dis Soc. 2023;12:222–5. 10.1093/jpids/piad003 36718660 PMC10146919

[11] Canadian Nosocomial Infection Surveillance Program. Health infobase: Canadian Nosocomial Infection Surveillance Program, 2024 [cited 2025 Dec 12]. https://health-infobase.canada.ca/cnisp/index.html

[12] Canadian Nosocomial Infection Surveillance Program1. Healthcare-associated infections and antimicrobial resistance in Canadian acute care hospitals, 2018-2022. Can Commun Dis Rep. 2024;50:179–96. 10.14745/ccdr.v50i06a02 39132584 PMC11315584

[13] Surveillance CNI. Healthcare-associated infections and antimicrobial resistance in Canadian acute care hospitals, 2014-2018. Can Commun Dis Rep. 2020;46:99–112. 10.14745/ccdr.v46i05a01 32558807 PMC7279130

[14] Infection Prevention and Control Canada. CNISP protocols & publications, 2024 [cited 2025 Jan 13]. https://ipac-canada.org/cnisp-publications

[15] Miller M, Gravel D, Mulvey M, Taylor G, Boyd D, Simor A, et al. Health care-associated *Clostridium difficile* infection in Canada: patient age and infecting strain type are highly predictive of severe outcome and mortality. Clin Infect Dis. 2010;50:194–201. 10.1086/649213 20025526

[16] Lynch T, Chong P, Zhang J, Hizon R, Du T, Graham MR, et al.; Canadian Nosocomial Infection Surveillance Program (CNISP). Characterization of a stable, metronidazole-resistant *Clostridium difficile* clinical isolate. PLoS One. 2013;8:e53757. 10.1371/journal.pone.0053757 23349739 PMC3547915

[17] Du T, Choi KB, Silva A, Golding GR, Pelude L, Hizon R, et al. Characterization of healthcare-associated and community-associated *Clostridioides difficile* infections among adults, Canada, 2015–2019. Emerg Infect Dis. 2022;28:1128–36. 10.3201/eid2806.212262 35470794 PMC9155897

[18] Fawley WN, Knetsch CW, MacCannell DR, Harmanus C, Du T, Mulvey MR, et al. Development and validation of an internationally-standardized, high-resolution capillary gel-based electrophoresis PCR-ribotyping protocol for *Clostridium difficile.* PLoS One. 2015;10:e0118150. 10.1371/journal.pone.0118150 25679978 PMC4332677

[19] Xia Y, Tunis MC, Frenette C, Katz K, Amaratunga K, Rose SR, et al. Epidemiology of *Clostridioides difficile* infection in Canada: A six-year review to support vaccine decision-making. Can Commun Dis Rep. 2019;45:191–211. 10.14745/ccdr.v45i78a04 31355824 PMC6615439

[20] Clinical and Laboratory Standards Institute. Performance standards for antimicrobial susceptibility testing of anaerobic bacteria, 9th edition (M11). Wayne (PA): The Institute; 2018.

[21] Ho J, Wong SH, Doddangoudar VC, Boost MV, Tse G, Ip M. Regional differences in temporal incidence of *Clostridium difficile* infection: a systematic review and meta-analysis. Am J Infect Control. 2020;48:89–94. 10.1016/j.ajic.2019.07.005 31387772

[22] Guh AY, Mu Y, Winston LG, Johnston H, Olson D, Farley MM, et al.; Emerging Infections Program Clostridioides difficile Infection Working Group. Emerging Infections Program *Clostridioides difficile* Infection Working Group. Trends in U.S. burden of *Clostridioides difficile* infection and outcomes. N Engl J Med. 2020;382:1320–30. 10.1056/NEJMoa1910215 32242357 PMC7861882

[23] Zilberberg MD, Tillotson GS, McDonald C. *Clostridium difficile* infections among hospitalized children, United States, 1997-2006. Emerg Infect Dis. 2010;16:604–9. 10.3201/eid1604.090680 20350373 PMC3363321

[24] Kim J, Smathers SA, Prasad P, Leckerman KH, Coffin S, Zaoutis T. Epidemiological features of *Clostridium difficile*-associated disease among inpatients at children’s hospitals in the United States, 2001-2006. Pediatrics. 2008;122:1266–70. 10.1542/peds.2008-0469 19047244

[25] Le Saux N, Gravel D, Mulvey M, Moore D, Langley JM, Richardson S, et al.; Canadian Nosocomial Infection Surveillance Program. Healthcare-associated *Clostridium difficile* infections and strain diversity in pediatric hospitals in the Canadian Nosocomial Infection Surveillance Program, 2007–2011. J Pediatric Infect Dis Soc. 2015;4:e151–4. 10.1093/jpids/piv011 26407250

[26] Silva A, Du T, Choi KB, Pelude L, Golding GR, Hizon R, et al.; CNISP C. difficile working group. CNISP C. difficile working group. Epidemiology of primary and recurrent healthcare-associated and community-associated pediatric *Clostridioides difficile* infection in Canada, 2015–2020. J Pediatric Infect Dis Soc. 2023;12:222–5. 10.1093/jpids/piad003 36718660 PMC10146919

[27] Pereira JA, McGeer A, Tomovici A, Selmani A, Chit A. Incidence and economic burden of *Clostridioides difficile* infection in Ontario: a retrospective population-based study. CMAJ Open. 2020;8:E16–25. 10.9778/cmajo.20190018 32001435 PMC7004222

[28] Bogaty C, Lévesque S, Garenc C, Frenette C, Bolduc D, Galarneau LA, et al.; Quebec Clostridium difficile Infection Surveillance Program (QCISP). Trends in the use of laboratory tests for the diagnosis of Clostridium difficile infection and association with incidence rates in Quebec, Canada, 2010-2014. Am J Infect Control. 2017;45:964–8. 10.1016/j.ajic.2017.04.002 28549882

[29] Kotila SM, Mentula S, Ollgren J, Virolainen-Julkunen A, Lyytikäinen O. Community- and healthcare-associated *Clostridium difficile* infections, Finland, 2008–2013. Emerg Infect Dis. 2016;22:1747–53. 10.3201/eid2210.151492 27648884 PMC5038409

[30] Tan XQ, Verrall AJ, Jureen R, Riley TV, Collins DA, Lin RT, et al. The emergence of community-onset *Clostridium difficile* infection in a tertiary hospital in Singapore: a cause for concern. Int J Antimicrob Agents. 2014;43:47–51. 10.1016/j.ijantimicag.2013.09.011 24290727

[31] Fellmeth G, Yarlagadda S, Iyer S. Epidemiology of community-onset *Clostridium difficile* infection in a community in the South of England. J Infect Public Health. 2010;3:118–23. 10.1016/j.jiph.2010.07.002 20869672

[32] Centers for Disease Control and Prevention. 2018 Annual report for the Emerging Infections Program for *Clostridioides difficile* infection. Atlanta: The Centers; 2019.

[33] European Centre for Disease Prevention and Control. *Clostridioides difficile* infections annual epidemiological report for 2019–2020. Stockholm; The Centre: 2024.

[34] Edwards PT, Thurm CW, Hall M, Busing JD, Kahn SA, Kellermayer R, et al. *Clostridioides difficile* infection in hospitalized pediatric patients: comparisons of epidemiology, testing, and treatment from 2013 to 2019. J Pediatr. 2023;252:111–116.e1. 10.1016/j.jpeds.2022.08.030 36027981 PMC9771922

[35] Chien M-M, Chang M-H, Chang K-C, Ni Y-H, Wu J-F. The incidence of *Clostridium difficile* infection in children with and without inflammatory bowel diseases: A single-center study in Taiwan from 2006 to 2019. J Formos Med Assoc. 2025;124:253–7. 10.1016/j.jfma.2024.04.006 38631957

[36] Stubbs SLJ, Brazier JS, O’Neill GL, Duerden BI. PCR targeted to the 16S-23S rRNA gene intergenic spacer region of *Clostridium difficile* and construction of a library consisting of 116 different PCR ribotypes. J Clin Microbiol. 1999;37:461–3. 10.1128/JCM.37.2.461-463.1999 9889244 PMC84342

[37] Carlson TJ, Blasingame D, Gonzales-Luna AJ, Alnezary F, Garey KW. *Clostridioides difficile* ribotype 106: A systematic review of the antimicrobial susceptibility, genetics, and clinical outcomes of this common worldwide strain. Anaerobe. 2020;62:102142. 10.1016/j.anaerobe.2019.102142 32007682 PMC7153973

[38] Roxas BAP, Roxas JL, Claus-Walker R, Harishankar A, Mansoor A, Anwar F, et al. Phylogenomic analysis of *Clostridioides difficile* ribotype 106 strains reveals novel genetic islands and emergent phenotypes. Sci Rep. 2020;10:22135. 10.1038/s41598-020-79123-2 33335199 PMC7747571

[39] Suárez-Bode L, Barrón R, Pérez JL, Mena A. Increasing prevalence of the epidemic ribotype 106 in healthcare facility-associated and community-associated *Clostridioides difficile* infection. Anaerobe. 2019;55:124–9. 10.1016/j.anaerobe.2018.12.002 30550807

[40] Jassem AN, Prystajecky N, Marra F, Kibsey P, Tan K, Umlandt P, et al. Characterization of *Clostridium difficile* strains in British Columbia, Canada: a shift from NAP1 majority (2008) to novel strain types (2013) in one region. Can J Infect Dis Med Microbiol. 2016;2016:8207418. 10.1155/2016/8207418 27366181 PMC4904575

[41] Karlowsky JA, Zhanel GG, Hammond GW, Rubinstein E, Wylie J, Du T, et al. Multidrug-resistant North American pulsotype 2 *Clostridium difficile* was the predominant toxigenic hospital-acquired strain in the province of Manitoba, Canada, in 2006-2007. J Med Microbiol. 2012;61:693–700. 10.1099/jmm.0.041053-0 22301615

[42] Public Health England. *Clostridium difficile* Ribotyping Network (CDRN) for England and Northern Ireland, 2015–2018. London: Public Health England; 2019.

[43] Keessen EC, Harmanus C, Dohmen W, Kuijper EJ, Lipman LJ. *Clostridium difficile* infection associated with pig farms. Emerg Infect Dis. 2013;19:1032–4. 10.3201/eid1906.121645 23735347 PMC3713831

[44] Knetsch CW, Kumar N, Forster SC, Connor TR, Browne HP, Harmanus C, et al. Zoonotic transfer of *Clostridium difficile* harboring antimicrobial resistance between farm animals and humans. J Clin Microbiol. 2018;56:e01384–17. 10.1128/JCM.01384-17 29237792 PMC5824051

[45] Peng Z, Jin D, Kim HB, Stratton CW, Wu B, Tang YW, et al. Update on antimicrobial resistance in *Clostridium difficile*: resistance mechanisms and antimicrobial susceptibility testing. J Clin Microbiol. 2017;55:1998–2008. 10.1128/JCM.02250-16 28404671 PMC5483901

[46] Wieczorkiewicz JT, Lopansri BK, Cheknis A, Osmolski JR, Hecht DW, Gerding DN, et al. Fluoroquinolone and macrolide exposure predict *Clostridium difficile* infection with the highly fluoroquinolone- and macrolide-resistant epidemic *C. difficile* strain BI/NAP1/027. Antimicrob Agents Chemother. 2015;60:418–23. 10.1128/AAC.01820-15 26525793 PMC4704185

[47] Tenover FC, Tickler IA, Persing DH. Antimicrobial-resistant strains of *Clostridium difficile* from North America. Antimicrob Agents Chemother. 2012;56:2929–32. 10.1128/AAC.00220-12 22411613 PMC3370774

[48] Freeman J, Vernon J, Morris K, Nicholson S, Todhunter S, Longshaw C, et al.; Pan-European Longitudinal Surveillance of Antibiotic Resistance among Prevalent Clostridium difficile Ribotypes’ Study Group. Pan-European longitudinal surveillance of antibiotic resistance among prevalent Clostridium difficile ribotypes. Clin Microbiol Infect. 2015;21:248.e9–16. 10.1016/j.cmi.2014.09.017 25701178

[49] Freeman J, Vernon J, Pilling S, Morris K, Nicolson S, Shearman S, et al.; Pan-European Longitudinal Surveillance of Antibiotic Resistance among Prevalent *Clostridium difficile* Ribotypes’ Study Group. Five-year Pan-European, longitudinal surveillance of Clostridium difficile ribotype prevalence and antimicrobial resistance: the extended ClosER study. Eur J Clin Microbiol Infect Dis. 2020;39:169–77. 10.1007/s10096-019-03708-7 31811507 PMC6962284

