# Supplementary material for: Characterization of Adult and Pediatric Healthcare-Associated and Community-Associated Clostridioides difficile Infections, Canada, 2015–2022
Source: Emerg Infect Dis. 2025 Jun;31(6):1109–20. doi: 10.3201/eid3106.250182 (PMC12123908; doi:10.3201/eid3106.250182)
Supplement: Appendix 1 — Additional information on characterization of adult and pediatric healthcare-associated and community-associated Clostridioides difficile infections, Canada, 2015–2022. [file 25-0182-Techapp-s1.pdf]

Article DOI: https://doi.org/10.3201/eid3106.250182

EID cannot ensure accessibility for supplementary materials supplied by authors. Readers who have difficulty accessing supplementary content should contact the authors for assistance.

Characterization of Adult and Pediatric Healthcare-Associated and Community-Associated *Clostridioides difficile* Infections, Canada, 2015–2022

Appendix 1

Appendix 1 Table 1. Number of Canadian Nosocomial Infection Surveillance Program hospitals in each region included in a study of *Clostridioides difficile* infection in adult and pediatric patients, Canada, 2015–2022*

Region	2015	2016	2017	2018	2019	2020	2021	2022
Adult (≥18 y)								
Healthcare-associated								
National	58	59	60	60	63	75	75	75
Western	22	22	22	22	22	23	23	23
Central	22	22	23	23	25	26	26	26
Eastern	14	15	15	15	15	25	25	25
Northern	0	0	0	0	1	1	1	1
Community-associated								
Western	47	48	50	50	53	63	63	63
Central	12	12	12	12	12	12	12	12
Eastern	21	21	23	23	25	26	26	26
Northern	14	15	15	15	15	24	24	24
Western	0	0	0	0	1	1	1	1
Pediatric (<18 y)								
Healthcare-associated								
Western	33	34	34	34	36	35	35	35
Central	16	16	16	16	16	16	16	16
Eastern	10	10	10	10	11	10	10	10
Northern	7	8	8	8	8	8	8	8
Western	0	0	0	0	1	1	1	1
Community-associated								
National	27	28	29	29	31	30	30	30
Western	11	11	11	11	11	11	11	11
Central	9	9	10	10	11	10	10	10
Eastern	7	8	8	8	8	8	8	8
Northern	0	0	0	0	1	1	1	1

*Western region includes British Columbia, Alberta, Saskatchewan, and Manitoba; Central includes Ontario and Quebec; Eastern includes Nova Scotia, New Brunswick, Prince Edward Island, and Newfoundland and Labrador; and Northern includes Nunavut.

Appendix 1 Table 2. Prevalence of adult and pediatric livestock-associated *Clostridioides difficile* ribotypes 078/126, Canada, 2015–2022

Ribotype 078/126	2015	2016	2017	2018	2019	2020	2021	2022	2015–2022
Adult, no. (%)	15 (2.6)	10 (2.1)	14 (2.7)	9 (1.8)	9 (2.0)	16 (5.8)	15 (3.5)	10 (2.9)	98 (2.8)
Pediatric, no. (%)	2 (1.2)	3 (1.6)	6 (3.8)	2 (1.5)	2 (1.7)	1 (1.1)	4 (3.5)	2 (2.2)	22 (2.1)

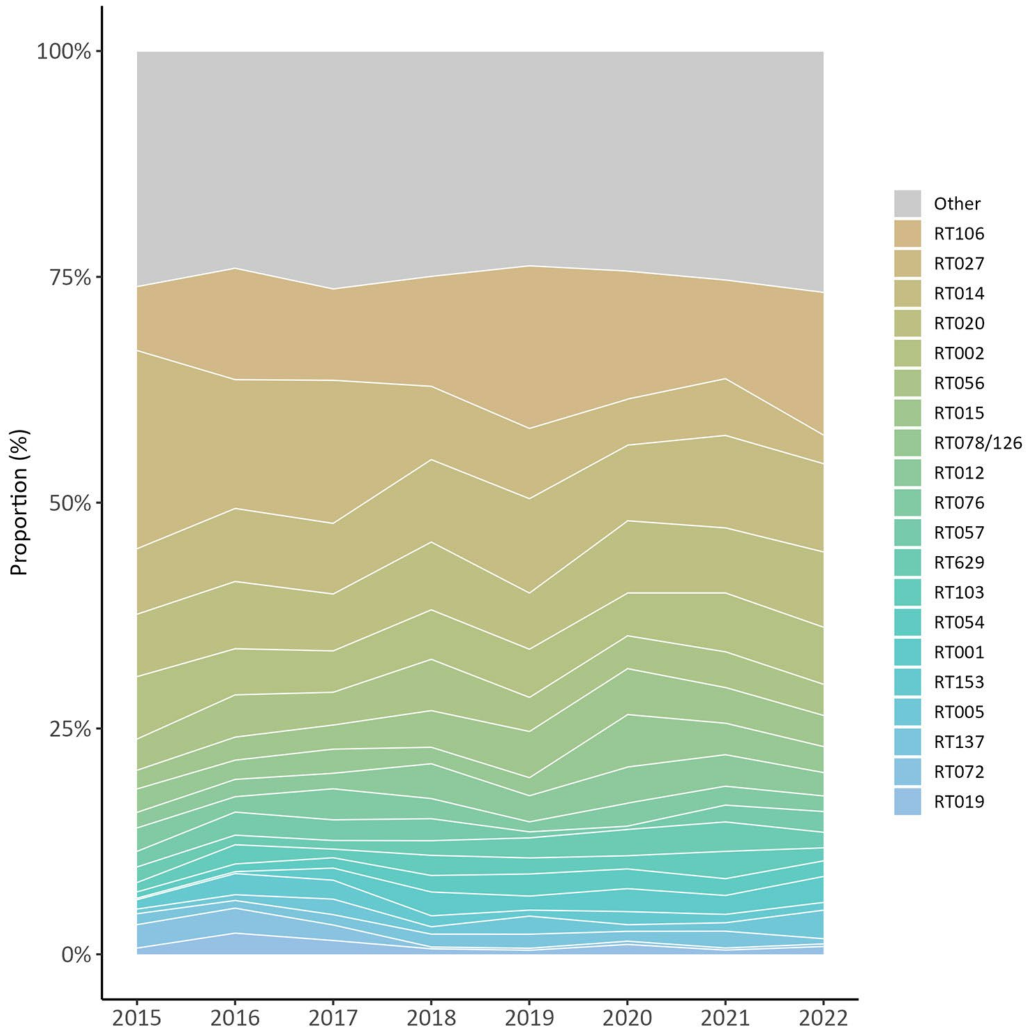

Appendix 1 Figure 1. Top 20 adult *Clostridioides difficile* ribotypes, Canada, 2015–2022.

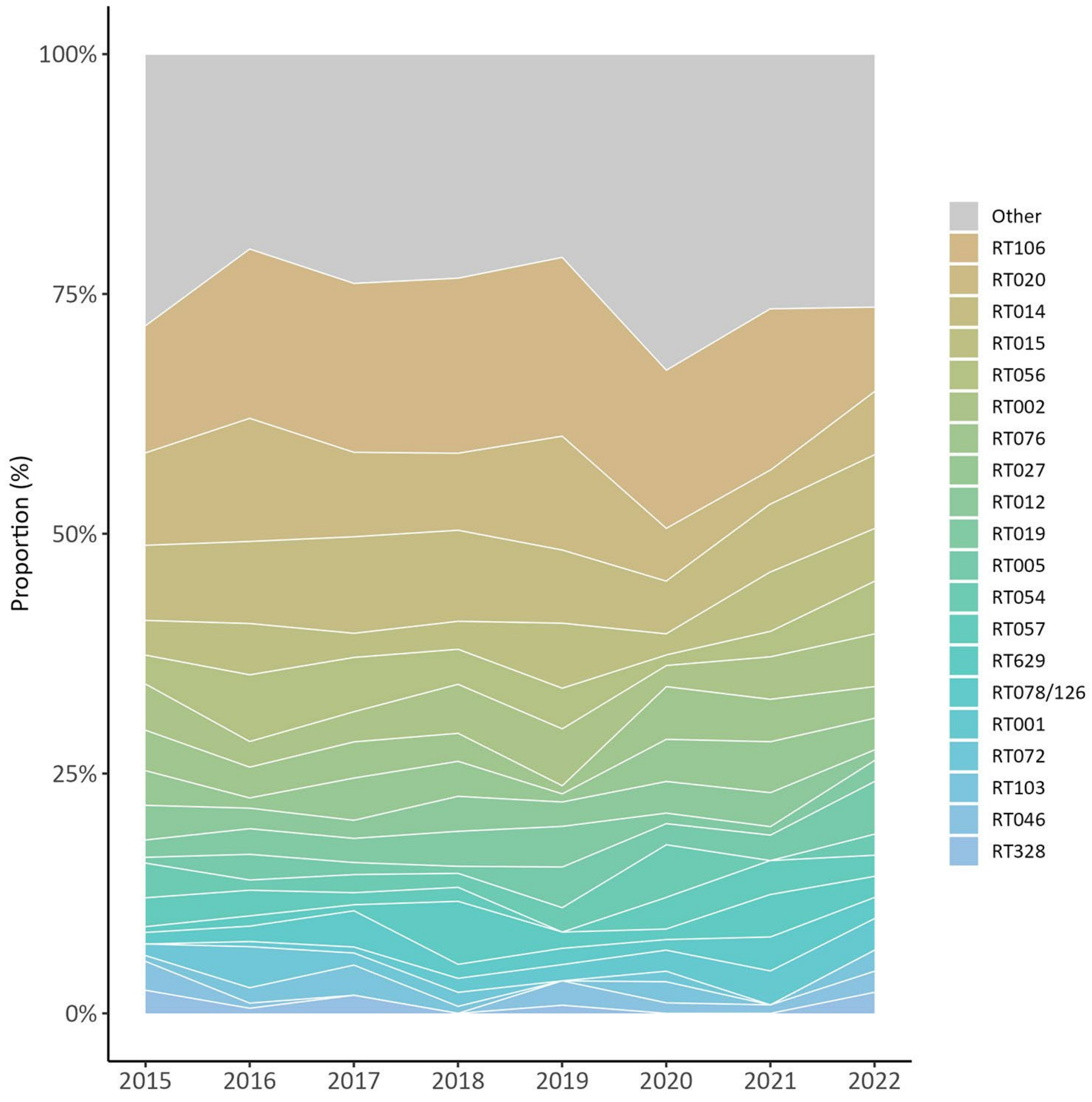

Appendix 1 Figure 2. Top 20 pediatric *Clostridioides difficile* ribotypes, Canada, 2015–2022.
